# SSD-EMB: An Improved SSD Using Enhanced Feature Map Block for Object Detection

**DOI:** 10.3390/s21082842

**Published:** 2021-04-17

**Authors:** Hong-Tae Choi, Ho-Jun Lee, Hoon Kang, Sungwook Yu, Ho-Hyun Park

**Affiliations:** School of Electrical and Electronics Engineering, Chung-Ang University, Seoul 06974, Korea; sksmsghdxo@cau.ac.kr (H.-T.C.); ghwns9817@cau.ac.kr (H.-J.L.); hkang@cau.ac.kr (H.K.); sungwook@cau.ac.kr (S.Y.)

**Keywords:** object detection, SSD, attention mechanism, feature map concatenation

## Abstract

The development of deep learning has achieved great success in object detection, but small object detection is still a difficult and challenging task in computer vision. To address the problem, we propose an improved single-shot multibox detector (SSD) using enhanced feature map blocks (SSD-EMB). The enhanced feature map block (EMB) consists of attention stream and feature map concatenation stream. The attention stream allows the proposed model to focus on the object regions rather than background owing to channel averaging and the effectiveness of the normalization. The feature map concatenation stream provides additional semantic information to the model without degrading the detection speed. By combining the output of these two streams, the enhanced feature map, which improves the detection of a small object, is generated. Experimental results show that the proposed model has high accuracy in small object detection. The proposed model not only achieves good detection accuracy, but also has a good detection speed. The SSD-EMB achieved a mean average precision (mAP) of 80.4% on the PASCAL VOC 2007 dataset at 30 frames per second on an RTX 2080Ti graphics processing unit, an mAP of 79.9% on the VOC 2012 dataset, and an mAP of 26.6% on the MS COCO dataset.

## 1. Introduction

Object detection is currently used in various applications, such as autonomous vehicles [1], face detection [2], medical imaging [3], and security [4]. Recently, the development of the convolutional neural networks (CNNs) concept [5,6,7,8] and the availability of large-scale datasets [9,10] have considerably improved the performance of object detection [11,12,13,14,15,16]. Researchers have expended numerous efforts to boost performance in various ways, such as optimizer design [17,18,19], modification of architecture [20], and scale variations [21,22] for computer vision. A fundamental approach used to effectively boost performance is based on the design of a good network. Since the introduction of the first deep neural network AlexNet [5] in 2012, various architectures have emerged, including the visual geometry group network (VGGNet) [6], GoogLeNet [7], and residual neural network (ResNet) [8]. With the development of graphics processing unit (GPU) power, these networks have yielded significant performance boosts by stacking the convolutional layers deeper, depending on their design choices. This allows us to obtain high-level semantic features that are extracted from the deep convolutional layers. These networks are being used in several computer vision models for applications such as object tracking [23], domain adaptation [24], and object detection.

The single-shot multibox detector (SSD) [15] has achieved high performance in detection accuracy and speed. The SSD algorithm was proposed to solve the problem of low-detection accuracy of the you-only-look-once (YOLO) model [14]. In SSD, the main goal is to detect multiple objects in an image. When detecting the objects, the object bounding box is predicted by prior boxes at different scales and aspect ratios followed by the generation of the feature map by CNN and the classification and regression of the objects in an image. The input image enters the network and then goes through the convolutional layers. Detection is performed using feature maps of different sizes in each of the six layers. The main idea of the SSD is to detect large objects based on the low-resolution feature maps of the deep layers, and detect small objects using the high-resolution feature maps of the shallow layers. The SSD detects large objects accurately, but its accuracy of small object detection is lower. We inferred that this problem is caused by two reasons, as described below.

First, in deep learning, the high-resolution feature maps of the shallow layers tend to contain fewer semantic information compared with the feature maps of the deep layers. This is attributed to the fact that the high-resolution feature maps pass through fewer convolutional layers compared with the feature maps of the deep layers. The methods employed to solve this problem in previous studies [25,26] involved the generation of a feature fusion module or the modification of the feature extraction network to develop an improved model. One of these studies [25] created the trident feature and squeeze and excitation feature fusion modules to add semantic information to the feature maps of the shallow layers. Another study [26] changed the network to DenseNet and used the residual prediction block. However, the problem associated with these methods relates to the detection speed degradation owing to the use of the complex network and the addition of complicated feature fusion modules. In order to extract more semantic information while concurrently maintaining the detection speed, we propose a lightweight feature map concatenation stream that splits the channels of the input feature map in half and performs three convolution operations on one of the half maps. Low-rank approximation is a representative method of compressing feature maps, which simplifies the network by reducing the parameter dimensions. Most methods [27,28] minimize the reconstruction error of original parameters to approximate a tensor. However, since these methods utilize the weight sparsity, they are useful when was applied to a limited resource environment such as a mobile platform, and the output feature map deviates far from the original values because errors tend to accumulate when multiple layers are compressed sequentially. Low-rank approximation has greatly improved the speed of CNNs, but it tends to decrease the accuracy. Also, the model cannot get a big gain in speed unless we apply it to the backbone of the SSD. However, our approach is to insert a module called enhanced map block (EMB) between the layers of the SSD, leaving the backbone of the SSD intact, therefore the low-rank approximation is not applied in our approach. Instead, we only use half of the weights of the feature map for convolutional layers to suppress the number of parameters increased by convolution. In this manner, the amount of learning is halved, compared to general convolution. Finally, the feature map that passed through the convolutional layers and the other feature map are concatenated with each other using skip connection.

Second, low-level features extracted from the shallow layers activate edges or background in an image. At this point, we have concluded that if the model can focus on an object, it can detect the region where small objects are captured. To execute this approach, we propose an efficient attention mechanism stream that employs the importance map. The importance map is produced by channel-wise average pooling and a sigmoid activation function. In this way, the pixels of the input feature map are averaged and then normalized to (0, 1). When generating the importance map, the model does not perform convolution operations. In this stream, the model only performs two simple operations with no additional learning. Therefore, it prevents degradation in the detection speed. Finally, we multiply the importance map and concatenated map element-wise, and add it to the input feature map. In this way, we propose the enhanced map block (EMB), which efficiently and effectively detects small objects. 

**Figure 1:**
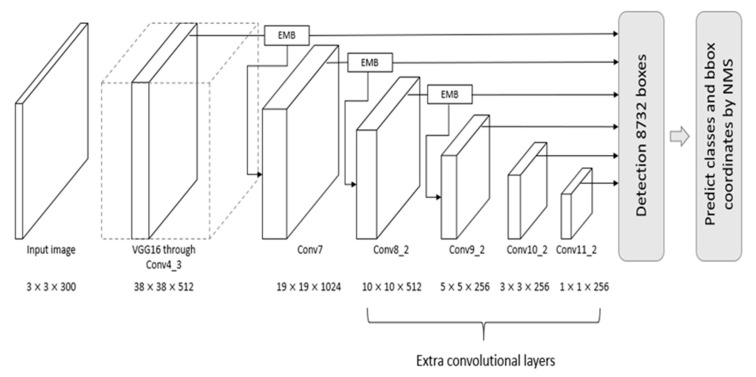
Overall architecture of the single-shot multibox detector with the enhanced map block (SSD-EMB). The input of the EMB is a feature map produced from convolutional layers. The EMB is applied after the Conv4_3, Conv7, and Conv8_2 layers. The output of the EMB is used as an input of the next convolutional layer. Please note that this figure presents the case in which the detector is the SSD300. If the SSD512 detector is used, an additional EMB is applied after Conv9_2. Similar to the original SSD, the 8732 bounding boxes include 5776 (38 × 38 × 4) boxes from Conv4_3, 2166 (19 × 19 × 6) boxes from Conv7, 600 (10 × 10 × 6) boxes from Conv8_2, 150 (5 × 5 × 6) boxes from Conv9_2, 36 (3 × 3 × 4) boxes from Conv10_2, and 4 (1 × 1 × 4) boxes from Conv11_2.

**Figure 2:**
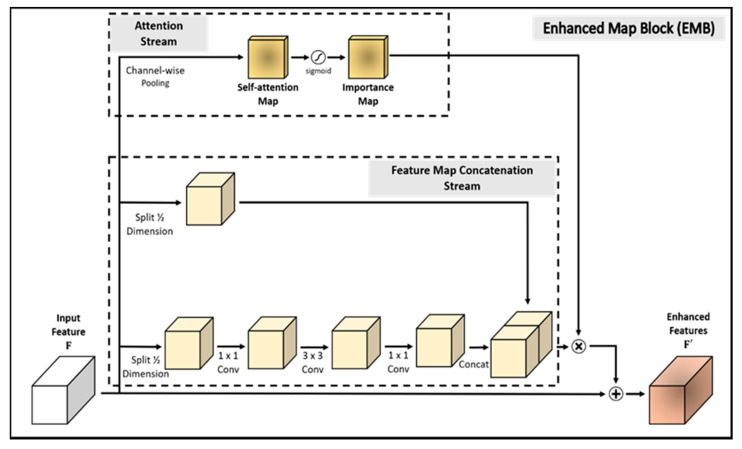
EMB block diagram. The self-attention map is produced by channel-wise average pooling on the input feature map generated by convolutional layers. Based on the self-attention map, we generate the importance map using a sigmoid activation function. The concatenation stream splits the feature map in half, performs three convolution operations on one of the half maps, and concatenates it with the other half.

In this study, an improved single-shot multibox detector (SSD) is proposed that employs novel and effective EMBs, called SSD-EMB. Although we used ideas from other methods, we created a new type of block, called the EMB, and applied it to the network to improve the accuracy without decreasing the detection speed. Object detectors using other types of feature map blocks have been studied. In [29], the feature extraction capability of the model was improved by integrating four inception blocks in an SSD. Each inception block consists of eight convolutional layers and a concatenation layer. Ding et al. [30] used four dense blocks in their SSD to enhance the features by integrating the features of the shallow layer and the features of the deep layer. These dense blocks contain eight convolutional layers and four concatenation layers, and they increase the network complexity and detection time. However, the proposed EMB is constructed more simply than other feature map blocks. We evaluate the efficiency of EMB on the detection task. Experimental datasets are the PASCAL visual object classes (VOC) 2007, the PASCAL VOC 2012 [31], and the MS COCO [10]. Our model is compared with the conventional object detection models, such as the faster region-based CNN (R-CNN) [13], YOLO [14], and SSD [15]. The overall architecture of SSD-EMB is shown in Figure 1. A detailed block diagram of the EMB is shown in Figure 2. The main contributions of this study are as follows:

- We propose a lightweight feature map concatenation stream, which consists of feature map split, three convolutional layers, and feature map concatenation.
- We present an efficient attention mechanism stream that applies channel-wise average pooling and sigmoid activation function on the input feature map.
- Combining the above two streams, the proposed model, SSD-EMB, solves the challenges associated with both small object detection and detection speed degradation.

We verify the effectiveness of the proposed model compared with various models (Faster R-CNN [13], YOLO [14], SSD [15], etc.) based on the PASCAL VOC 2007, PASCAL VOC 2012, and MS COCO datasets. Our model detects objects with high accuracy and speed, and moreover, it effectively captures small objects.

The rest of this study is organized as follows: Section 2 is a description of the related research. Section 3 describes the proposed approach. Section 4 shows the results of conducted experiments with the datasets and compares them to other models. The conclusions are listed in Section 5.

## 2. Related Work

### 2.1. Attention Mechanism

The attention mechanism has recently been actively used in various fields, such as machine translation [32], image inpainting [33], image captioning [34], and generative models [35]. Attention is a technique that allows an artificial model to focus on semantic features rather than the entire features equally. In general, it is called self-attention, and its purpose is to effectively learn the meaningful representation of the data to perform certain tasks.

Recently, various studies [36,37,38,39,40] have used the self-attention mechanism to improve the classification accuracy. Hu et al. [36] proposed the squeeze-and-excitation (SE) block that increased the accuracy of the classification model based on the use of a one-dimensional (1D) channel self-attention map. Wang et al. [37] formulated self-attention as a non-local operation, covering the entire image region in one operation to model spatial-temporal dependencies in video sequences. Park et al. [38] and Choe et al. [40] proposed the bottleneck attention module (BAM) and attention-based dropout layer (ADL) that respectively produced spatial self-attention and importance maps with auxiliary convolutional layers. The produced self-attention map is applied to the input feature map to emphasize the object region. Similarly, the proposed method generates the importance map for capturing the important region.

There are several studies that have used the attention mechanism for object detection. Gao et al. [41] proposed the instance-centric attention network (iCAN), which generates an attention map using the appearances of humans and objects and applied it to the feature map to obtain attention-based contextual features. Carion et al. [42] proposed a detection transformer, called DETR, that employs an encoder and decoder for object detection. They used it to remove redundant predictions through the self-attention layers of the encoder–decoder structure of the transformer. Ning et al. [43] used an attention mechanism for person re-identification. To identify high value features and eliminate interference caused by background information, they designed a multibranch attention network to select valuable fine-grained features.

### 2.2. Single-Shot Multibox Detector (SSD)

The models that have been extensively used as one-stage detectors include the YOLO [14] and SSD [15]. YOLO considers the bounding boxes and class probability in an image as a single regression problem. By looking at the image once, it predicts the class and the location of the object simultaneously. This model has low accuracy as it is designed to perform detection using only the last layer. Since the introduction of the first version of YOLO, YOLO:9000 [44], YOLOv3 [45], and YOLOv4 [46] have also been proposed. YOLO:9000 introduced an optimal anchor box by clustering bounding boxes to detect objects. YOLOv3 improved the performance by changing the feature extractor and matching of the bounding box with the highest intersection-over-union (IoU) with the ground-truth box. YOLOv4 can be used on a single GPU and was designed for implementation in real-time tasks. The authors utilized various state-of-the-art methods, such as weighted-residual connection (WRC) and cross-stage-partial connection (CSP), to balance speed and accuracy.

The SSD uses VGG [6] as the backbone network and some auxiliary convolutional layers are added. Based on SSD300 with the input size of 300×300, a total of six feature maps with the layers of Conv4_3, Conv7, Conv8_2, Conv9_2, Conv10_2, and Conv_11_2 are independently employed to predict the object class and coordinates. Finally, it employs a non-maximum suppression (NMS) method for final detection. In this way, it is possible to detect objects at various scales using the feature map of each layer, thereby achieving better accuracy compared with the conventional methods. However, this model has a problem in detecting small objects because the feature maps of the shallow layers do not contain abundant high-level semantic information.

### 2.3. SSD-Based Object Detectors

To solve the above problem, several researchers have proposed the following models. The deconvolutional SSD (DSSD) [16] was proposed, which adds deconvolution operations to the end of SSD to utilize contextual information. Through the deconvolutional layer, contextual information can be passed to the shallow layer, which leads to an accuracy improvement in small object detection as the size of the feature map increases. However, the addition of the DSSD’s deconvolutional layers leads to computational complexity and additional parameter overhead.

Zhai et al. [26] proposed an SSD based on DenseNet and feature fusion (DF-SSD). The proposed model changes the backbone network for feature extraction from VGG to DenseNet-S-32-1 and uses a front-end-network, including a feature fusion module and a residual prediction module for object detection. In this way, DF-SSD uses a more powerful backbone and improves the accuracy by replacing the feature maps of Conv4_3, Conv7, Conv8_2, and Conv9_2 with Conv4_Fu, Conv7_Fu, Conv8_Fu, and Conv9_Fu.

The SSD using trident and squeeze and extraction feature fusion (SSD-TSEFFM) [25], which adds the trident feature module (TFM) and the squeeze and excitation feature fusion module (SEFFM), detects small objects effectively. TFM applies dilated convolution to make the proposed model robust to scale changes based on the consideration of the scale diversity. SEFFM is used to provide more semantic information to the model. Through these two modules, a new feature map is created with a focus on the important features. However, this model has a problem in that the detection speed is slowed down owing to an increase in the computational workload because of the use of additional modules. Unlike these methods, the proposed method not only improves the detection accuracy with fewer operations, but it also maintains the detection speed.

### 2.4. Other Object Detectors

Recently, various object detectors, apart from SSD-based detectors, have been studied. Among them, one-stage object detectors are of interest here. Tan et al. [47] proposed EfficientDet, which combines the weighted bidirectional feature network (BiFPN) with EfficientNet and compound scaling proposed in their previous work. Thus, they designed an efficient model by allowing the network to learn the valuable features of different input feature maps.

Dai et al. [48] proposed a new customized loss function and object detection model for power line area recognition and risk object detection in smart power surveillance systems. A loss function was designed by introducing a self-adaptive weight and a global weight as an object size factor to improve the accuracy for small objects, and an object detection model with competitive processing speed was proposed using a concise deep neural network model.

Qayyum et al. [49] acquired spatial data for high voltage power poles, urban areas, and vegetation near power lines using unmanned aerial vehicles (UAVs) and proposed a fuzzy-based classifier to classify small objects in the data. For object classification, they produced three decision rules based on the spectrum and color in UAV images.

### 2.5. Low-Rank Approximation of Feature Map

Several studies have been conducted to speed up the network by compressing the convolutional layers. Denton et al. [27] compressed each convolutional layer by finding a suitable low-rank approximation and then fine-tuned the upper layers until the prediction accuracy was restored. They considered some basic tensor decomposition based on singular value decomposition and the filter clustering method to utilize the similarity between learned features. Thus, the parameter redundancy of the network was exploited with linear compression, resulting in significant speedups of the trained network.

Jaderberg et al. [28] approximated a combination of a low rank basis of rank-1 filters in the spatial domain by exploiting the redundancy of cross-channel or the filter. They have drastically speeded up the convolutional layers, regardless of the architecture.

Ciccone at el. [50] reconstructed the sparse plus low-rank approximation problem for cases where only the sample covariance is available and the difference between the sample covariance and the actual covariance is not negligible. This has alleviated the problem of rapid degradation of results when the covariance matrix must be estimated from the observed data and is affected by certain degrees of uncertainty.

However, when low-rank approximation methods are applied, reconstruction errors due to compression occur, resulting in a loss of accuracy. To achieve a large speed boost, it should be applied to the backbone and extra convolutional layers of the original SSD. Our goal is to prevent the reduction in speed while improving the accuracy through a module, called the EMB, without modifying the backbone of the SSD. Therefore, we employed a feature map split and skip connection without low-rank approximation to reduce the learning parameters and prevent the degradation of detection speed of the original SSD.

## 3. Materials and Methods

In this section, we describe the proposed method in detail and introduce the evaluation datasets, metrics, and implementation details of the proposed model. The overall architecture of SSD-EMB and block diagram of EMB are shown in Figure 1 and Figure 2, respectively. Given an image, features are extracted by VGG [6] and are sent to auxiliary convolutional layers. The six feature maps of different resolutions are generated in the layers of Conv4_3, Conv7, Conv8_2, Conv9_2, Conv10_2, and Conv11_2. The SSD utilizes these features independently to predict the classes and locations of objects. We add an EMB to the Conv4_3, Conv7, and Conv8_2 feature maps of the SSD300 to detect small objects, as shown in Figure 1, and one more EMB to the Conv9_2 on the SSD512. Based on this approach, the feature maps, which applied the EMB, are used to obtain the final detection results by NMS. In this way, the feature map of the shallow layer includes the more semantic features, and small objects are accurately captured. The proposed method is constructed based on the attention stream and feature map concatenation stream. The former compresses the n-dimensional input feature map by channel-wise average pooling to produce the 1D self-attention map. The latter divides the channels of the input feature map in half, and executes the convolutions, and performs concatenation through skip connection. Feature maps enhanced by EMB are used as inputs to the subsequent convolutional layers of the network. By applying our block, we can efficiently detect small objects which can improve detection performance.

### 3.1. Attention Stream

**Figure 3:**
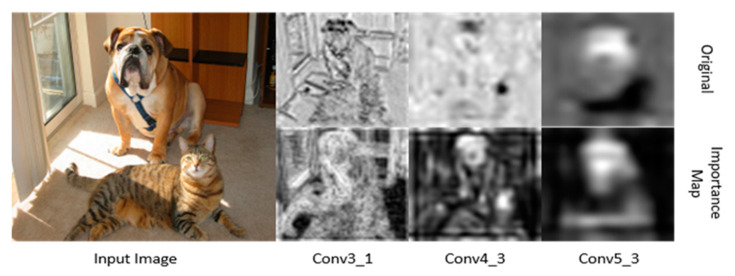
Original feature map and importance map at each visual geometry group-16 (VGG-16) layer. At the shallow layers, the feature map includes global features, whereas class-specific fea-tures are included at the deep layers. The importance map activates more regions with objects. We visualize that a pixel value the close to 0 is black, and close to 1 is white. Please note that the self-attention map has a similar distribution to the importance map. Thus, we do not show it.

In general, the high-resolution feature map of the shallow layer contains low-level features, as shown in the top of Conv3_1 of Figure 3. However, the low-resolution feature map of the deep layer has high-level semantic features as shown in the top of the Conv5_3 of Figure 3. The SSD employs three low-resolution feature maps and three high-resolution feature maps for object detection. In our model, the attention mechanism is used to focus the region of the object of the high-resolution feature maps activated by low-level features.

Specifically, in SSD300, EMB is applied to the feature map $f\in {\mathbb{R}}^{H\times W\times C}$ on the Conv4_3, Conv7, Conv8_2 layers, as shown in Figure 1. Note that C is the number of channels, and H and W are the height and width, respectively. First, the attention stream compresses all the channels of f by channel-wise average pooling, which sums all the pixels in the channels, and divides them by the number of channels to generate the self-attention map $m_{attn}\;\in \;{\mathbb{R}}^{H\times W\times 1}$. By averaging the pixel values of channels of the input feature map, a pixel with a high value is regarded as a high-level semantic feature. The produced map activates the object and the region around it. The importance map $m_{imp}\;\in \;{\mathbb{R}}^{H\times W\times 1}$ is then produced by calculating a sigmoid activation function as shown in Figure 2. In this way, the pixels in the importance map are normalized to (0, 1), smoothing out the transition of the pixel values of the feature map. Each pixel of the importance map close to 1 is regarded as the most discriminative part (foreground), while the pixel values close to 0 correspond to the less discriminative part (background). With these operations, the detector focuses on the object region. In brief, the importance map is computed as follows:

(1) $$m_{imp}=sigmoid\left(AvgPool\left(f\right)\right).$$

This component is inspired by [40]. The attention stream simply averages pixels of channels and computes a sigmoid activation function. The importance map increases the localization accuracy of the model by a broader activation of the region where the object is located.

### 3.2. Feature Map Concatenation Stream

In general, the choice used to obtain more semantic information in the CNN-based models is to stack the convolutional layers deeper. Convolutional operation requires a lot of computation as the number of weights increases, which leads to the network complexity and to a slower detection speed. To address this problem, we propose to extract semantic features using only half weights of the input feature map, as shown in Figure 2. To suppress the number of parameters increased via convolution, we split the input feature map in half to reduce the computational features and utilized a skip connection structure of ResNet. Therefore, the feature map concatenation stream splits the dimensions of the input feature map in half. One of the split feature maps $f_{\frac{1}{2}}\in {\mathbb{R}}^{H\times W\times \frac{C}{2}}$ passes through three convolutional layers to extract more semantic features. Because the model uses half of the weights, the computational amount is reduced compared with the general convolutional operation. During this operation, there is no change in the feature map size. In the convolution operation, batch normalization and ReLU activation function are also performed. Finally, the other ${f^{\prime }}_{\frac{1}{2}}\in {\mathbb{R}}^{H\times W\times \frac{C}{2}}$ is concatenated with the feature map $f_{\frac{1}{2}}$ that has passed through the convolutional layers, as shown in Figure 2. In brief, the concatenated feature map $m_{concat}\in {\mathbb{R}}^{H\times W\times C}$ is computed as follows:

(2) $$m_{concat}=concat\left(C_{2}^{1\times 1}\left(C_{1}^{3\times 3}\left(C_{0}^{1\times 1}\left(f_{\frac{1}{2}}\right)\right)\right),{f^{\prime }}_{\frac{1}{2}}\right),$$

where C denotes a convolutional operation, concat denotes a concatenation operation, $f_{\frac{1}{2}},\;{f^{\prime }}_{\frac{1}{2}}$ are the split feature maps, respectively, and the superscripts denote the convolutional filter sizes. Generating a feature map in this stream is possible using half of the original convolution operation. We experimentally verified that splitting the input feature map in half is the most efficient approach, considering the trade-off between accuracy and speed. In this way, the model can efficiently detect small objects with preventing degradation in the detection speed.

### 3.3. Combination of Two Maps

After acquiring the importance map $m_{imp}$ and feature map concatenation $m_{concat}$, we combine them to produce the final enhanced feature map $f^{\prime }\in {\mathbb{R}}^{H\times W\times C}$. The importance map $m_{imp}$ acts as an object-aware mask and is element-wise multiplied by the concatenated feature map $m_{concat}$ to generate an object-aware feature map. The final enhanced map $f^{\prime }$ is created by adding the object-aware feature map and the original input feature map according to Equation (3). Note that the enhanced feature map $f^{\prime }$ is used as an input of the next convolutional layer to share higher-level features compared with the existing model:

(3) $$f^{\prime }=f+(m_{concat}⨂m_{imp})$$

In this way, the SSD-EMB efficiently and effectively captures small objects. The EMB improves the detection accuracy and averts the detection speed of the model with simple extra computations. In addition, the proposed method can be plugged independently into each of the convolutional feature maps of the shallow layers.

### 3.4. Datasets and Evaluation Metrics

Two challenging and extensively used benchmark datasets in object detection, i.e., PASCAL VOC 2007 and 2012, are chosen to evaluate the proposed model. The PASCAL VOC datasets include 20 object categories. The PASCAL VOC 2007 and 2012 consist of 9963 and 22,531 images, respectively. For PASCAL VOC 2007, we used the VOC 2007 and 2012 trainval split (5011 images for 2007 and 11,540 images for 2012) to train our network and employed the test split (4952 images) for testing. To evaluate our model in VOC 2012, the model was trained with a total of 21,503 images of VOC 2007 trainval + test (9963 images) and VOC 2012 trainval (11,540 images). The proposed model was tested with the VOC 2012 test set (10,991 images).

We adopted the mean average precision (mAP) as the standard metric for object detection to evaluate our model in the test set. The metrics were obtained in accordance with the PASCAL VOC criterion in which the bounding box of a positive detection had an IoU > 0.5 with the ground truth annotation.

Finally, our model was tested on the MS COCO dataset. MS COCO consists of a total of 80 object categories and includes 118 k train images, 5 k validation images, and 20 k test images (test-dev). We used the train set for training and evaluated the detection results on test-dev 2015. Compared with PASCAL VOC, the MS COCO dataset contains more objects (small or general) in a single image. Therefore, COCO detection is a more difficult task.

### 3.5. Implementation Details

We adopted the SSD as our baseline detector. To evaluate the effectiveness in the same environment as the original SSD, our model also used the pre-trained VGG [6] as a backbone. In this study, most training strategies, including data augmentation and optimization, follow the ones in [15]. We plugged the EMB behind the convolutional layers. Our model was trained with 120,000 iterations for PASCAL VOC 2007. The batch size was set to 32 for SSD300 and SSD512 for PASCAL VOC 2007 and VOC 2012 based on consideration of our GPU specifications. The learning rate was $10^{-3}$ in the first 80,000 iterations, $10^{-4}$ in the next 20,000 iterations, and $10^{-5}$ in the remaining iterations in VOC 2007. In the VOC 2012 dataset, the number of training iterations increased to 150,000 because the amount of training images increased. The learning rate was $10^{-3}$ in the first 60,000 iterations, $10^{-4}$ in the next 60,000 iterations, and $10^{-5}$ in the remaining iterations. In the MS COCO dataset, the number of training iterations increased to 300,000 and the learning rate was $10^{-3}$ in the first 160,000 iterations, $10^{-4}$ in the next 40,000 iterations, $10^{-5}$ in the next 40,000 iterations, and $10^{-6}$ in the remaining iterations.

The entire network was optimized using stochastic gradient descent (SGD) with a momentum of 0.9 and a weight decay of 0.0005. In the training process, the proposed EMB was applied to the output of the three convolutional layers to capture small objects well, and other methods, such as data augmentation, were applied in the same manner as the original SSD. Other than those, there is no special treatment for small object detection alone. The loss function is a weighted sum of the localization loss (loc) and the confidence loss (conf). In object detection, the neural network must perform two tasks. We organize the two losses into a weighted sum so that one loss function does not wield too much influence. This means that if the localization loss is too large, the learning will be focused on localizing the object and the classification task will be vulnerable. We adjust this situation by defining the loss function as the form of a weighted sum. The weight term α is set to 1 by cross-validation. As in the other object detection literatures [15,26], the localization loss calculates the error between the predicted bounding box by our model and the ground-truth bounding box to determine how much the two boxes match, and the confidence loss calculates the error between the predicted object class and the ground-truth object class to determine how well our model classifies the object class. The equations below also adhere to the formulation described in [15]:

(4) $$L\left(x,\;c,\;l,\;g\right)=\frac{1}{N}\left(L_{conf}\left(x,c\right)+\alpha L_{loc}\left(x,l,g\right)\right),$$

where N is the number of positives of the default boxes, that is, it is the default box that can be matched with a ground-truth box. If objects are accurately predicted, it is treated as positives and the rest as negatives. Default boxes refer to boxes extracted from multiple feature layers of different scales. If N=0, we set the loss to 0. The localization loss was Smooth L1 loss between the predicted box (l) and the ground-truth box (g) parameters; thus, it is a curve when |x|<1. Therefore, if the error is small enough, it is judged to be almost correct and the loss decreases quickly. Similar to Faster R-CNN, we regressed to offsets for the center (cx, cy) of the default bounding box (d) and for its width (w) and height (h):

(5) $$L_{loc}\left(x,l,g\right)=\overset{N}{\underset{i\in Pos}{\sum }}\underset{m\in \left\{cx,cy,w,h\right\}}{\sum }x_{ij}^{k}smooth_{L1}\left(l_{i}^{m}-{\hat{g}}_{j}^{m}\right)$$

(6) $${\hat{g}}_{j}^{cx}=\left(g_{j}^{cx}-d_{i}^{cx}\right)/d_{i}^{w}\;\;\;\;\;\;\;\;\;\;{\hat{g}}_{j}^{cy}=\left(g_{j}^{cy}-d_{i}^{cy}\right)/d_{i}^{h}$$

(7) $${\hat{g}}_{j}^{w}=log\left(\frac{g_{j}^{w}}{d_{i}^{w}}\right)\;\;\;\;\;{\hat{g}}_{j}^{h}=log\left(\frac{g_{j}^{h}}{d_{i}^{h}}\right),$$

where

(8) $$smooth_{L1}\left(x\right)=\{\begin{array}{c} 0.5{\left(x\right)}^{2}\;\;\;\;\;\;\;\;\;\;\;\;\left|x\right|<1\\ \left|x\right|-0.5\;\;\;\;\;\;\;\;otherwise \end{array}.$$

The confidence loss is the softmax loss over multiple classes confidences (c) given by:

(9) $$L_{conf}\left(x,c\right)=-\overset{N}{\underset{i\in Pos}{\sum }}x_{ij}^{p}log\left({\hat{c}}_{i}^{p}\right)-\underset{i\in Neg}{\sum }log\left({\hat{c}}_{i}^{0}\right),$$

where

(10) $${\hat{c}}_{i}^{p}=\exp\left(c_{i}^{p}\right)/\underset{p}{\sum }\exp\left(c_{i}^{p}\right).$$

In SSD300, EMB was added after the Conv4_3, Conv7, Conv8_2 layers. In SSD512, EMB was added after the Conv4_3, Conv7, Conv8_2, Conv9_2 layers. Our model was implemented using the PyTorch deep learning framework [51] and executed on an Intel Xeon E5-2620V, Nvidia RTX 2080Ti GPU (Santa Clara, CA, USA). The source code is available at https://github.com/HTCho1/SSD-EMB.Pytorch/ (accessed on 14 April 2021).

## 4. Results and Discussion

### 4.1. Results on PASCAL VOC 2007 Test Set

The results of our method on PASCAL VOC 2007 are presented in Table 1. The input sizes of the proposed model are 300×300 and 512×512, respectively. In the model with an input size of 300×300, the SSD-EMB achieved a mAP of 78.4%, which is 1.2% points higher than the original SSD300. The models with the higher mAP were DSSD321 and SSD300-TSEFFM, but the detection speed dropped owing to the operation of additional modules as presented in Table 2. In the case of the model with the input size raised to 512×512, our model improved by 0.9% point compared with the SSD512. The overall of our model is 1.6% and 1.4% less than RefineDet [52] at input sizes 300 and 500, respectively. It is because the original SSD’s inherent structure is inferior to that of these models. Although the overall mAP of SSD-EMB is lower than that of RefineDet, SSD-EMB300 achieves a better accuracy on the following object classes: “bus,” “diningtable,” and “sofa”, and SSD-EMB512 is superior on “boat,” “chair,” “diningtable,” and “dog”. The subsequent version RefineDet++ [53] is better than RefineDet. However, the detailed mAP of each object was not mentioned in the paper; thus, it is excluded from Table 1.

The proposed model achieved an accuracy of >80% in classes such as “bus,” “car,” “cat,” and “dog.” Conversely, it yielded accuracies less than 60% for objects such as “bottle,” and “plant.” We can see that our method is higher than the original SSD300 in most object classes and exceeds by 1.2% on the overall mAP. Our model improved the accuracy by more than 2% on rigid objects (e.g., 50.8% vs. 54.2% mAP for “bottle,” 85.1% vs. 87.4% mAP for “bus,” 83.2% vs. 85.7% mAP for “train,” etc.), and on some nonrigid objects (e.g., 80.9% vs. 82.8% mAP for “cow” and 73.5% vs. 76.9% mAP for “sheep”). This is the result obtained by creating the object-aware mask in EMB to capture more accurate object regions and improve the detection accuracy, as shown in Figure 3. SSD300-EMB improves the accuracy of small objects because EMB focuses on the object regions and yields more semantic features. However, it is difficult for the detector to predict the object when part of an object is occluded. If only part of the object is visible in the image, the detector has a low accuracy.

Unlike other methods based on the SSD, SSD300-EMB has improved detection accuracy and preserved the FPS of the original SSD300. As listed in Table 2, the SSD300 achieved an mAP of 77.2% and 30 frames per second (FPS), and SSD300-EMB surpassed an mAP of 1.2% and maintained a rate of 30 FPS. SSD300*, achieved an mAP of 77.2% and 46 FPS, and the DF-SSD300 improved the accuracy by 1.4% and reduced the speed by more than four times. The SSD-based methods, such as DF-SSD300, SSD300-TSEFFM, DSSD321 improve the detection accuracies but the detection speeds are significantly slower than their baseline. However, we emphasize that the SSD300-EMB yields a similar improvement in detection accuracy to the SSD300-TSEFFM [25], DSSD321 [16] and DF-SSD300, but detection speed remains the same as SSD300. Therefore, the SSD-EMB can be used in real-time detection.

**Figure 4:**
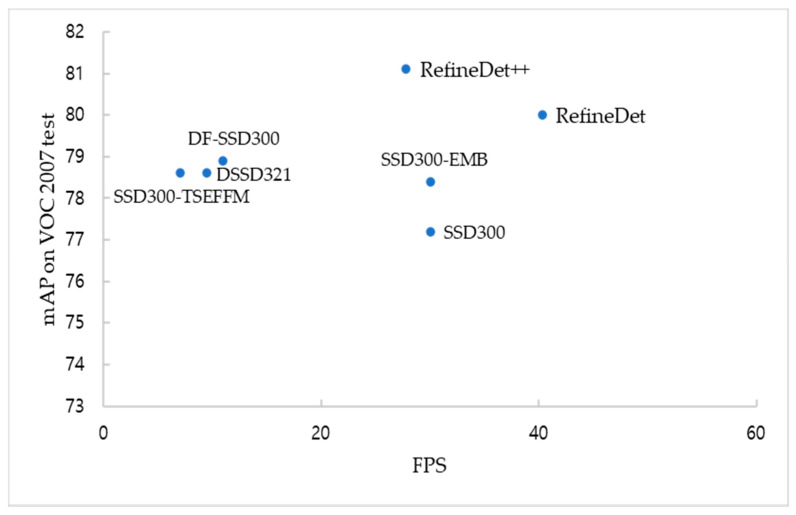
Distribution of speed and accuracy with object detection algorithms.

**Figure 5:**
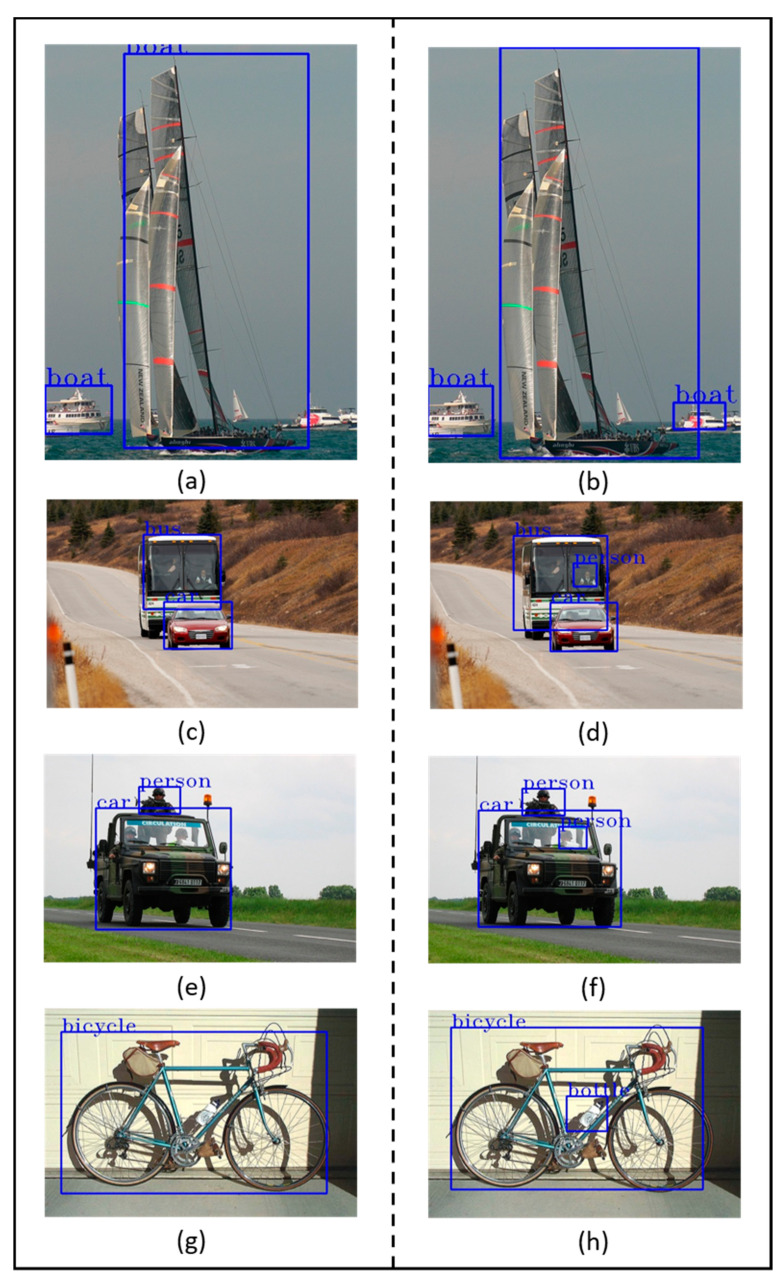

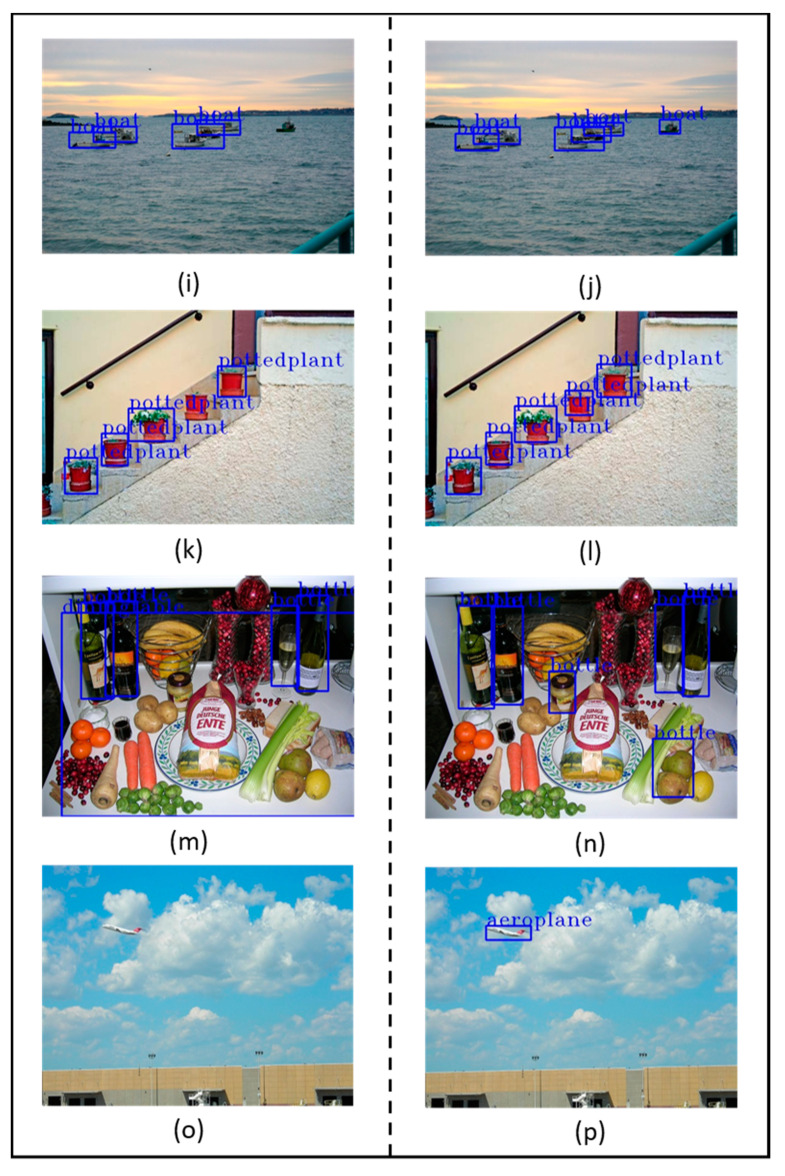
Examples of predicted bounding boxes generated by the baseline model and our proposed model tested on the PASCAL visual object classes (VOC) dataset. Left column images (**a**,**c**,**e**,**g**,**i**,**k**,**m**,**o**) show the results of SSD (i.e., choosing the highest score proposal as the pseudo-ground truth). Right column images (**b**,**d**,**f**,**h**,**j**,**l**,**n**,**p**) show some bounding boxes produced by our method (i.e., applying EMBs). Applying EMB to SSD to detect objects yields better outcomes than the original SSD in almost all cases. However, as shown in (**n**), objects with similar colors and appearances are sometimes incorrectly predicted.

The detection accuracy and speed distribution of different object detection algorithms on the VOC 2007 test set are shown in Figure 4. It can be observed that the SSD300-EMB yields a similar detection accuracy but a significantly faster speed than other SSD-based models. Compared with SSD300, the detection accuracy is improved while the speed does not decrease. In addition, some examples of the predicted bounding boxes generated by the SSD300 and SSD300-EMB are presented in Figure 5.

### 4.2. Results on PASCAL VOC 2012 Test Set

We conducted experiments on the PASCAL VOC 2012 dataset. The results are presented in Table 3. The proposed model was also tested using input images with sizes of 300×300 and 512×512. For the input size of 300×300, SSD300-EMB achieved an mAP of 77.0%, which is 1.2% points higher than the original SSD300. The accuracy of SSD300-EMB is 0.7% higher than DSSD321 and 0.5% higher than DF-SSD300. In the case of the model with an input size increased to 512×512, the SSD512-EMB was 79.9%, which was 1.4% points higher than the SSD512.

The SSD300-EMB and SSD512-EMB achieved accuracies of >80% in 11 and 12 classes, respectively. The SSD300-EMB showed improved accuracy in all classes except the “dog” class compared with the original SSD300. The “bicycle,” “bottle,” “table,” and “sofa” classes improved by more than 2% (e.g., 82.9% vs. 85.4% mAP, 47.6% vs. 50.3% mAP, 64.1% vs. 66.2% mAP, 73.6% vs. 75.6% mAP, respectively). For the input size of 512×512, the SSD512-EMB also achieved a higher mAP in most classes compared with the original SSD512. We designed the EMB to efficiently detect small objects. This led to an improved detection accuracy and preserved speed response compared with the original SSD. As shown in Table 3, the model with the highest accuracy is RefineDet. However, our model achieved better results for some rigid object classes such as “bicycle,” “bus,” “chair,” “diningtable,” “motorbike,” and “sofa” (e.g., 86.8% vs 88.5% mAP, 84.9% vs 86.2% mAP, 62.0% vs 62.7% mAP, 64.9% vs 67.1% mAP, 87.2% vs 89.5% mAP, and 72.5% vs 73.0% mAP respectively) and some non-rigid object classes such as “cat,” “dog,” and “horse” (e.g., 92.2% vs 93.1% mAP, 90.6% vs 91.3% mAP, and 88.3% vs 88.9% mAP, respectively).

### 4.3. Results on Microsoft Common Objects in Context (MS COCO)

Finally, the proposed model was evaluated on the MS COCO dataset. Since a large number of COCO objects tend to be smaller than PASCAL VOC, we set smaller prior boxes for all layers. The test results of SSD-EMB on test-dev 2015 are presented in Table 4. Our model, which improved the original SSD, achieved 26.6/45.2%. We note that our [0.5:0.95] result is 1.5% points higher than SSD300 (26.6% vs 25.1%) and small object detection result is 0.7% point higher than SSD300 (7.3% vs 6.6%).

However, the proposed model achieved lower accuracy than DSSD321 and DF-SSD300 (1.4% and 2.9% on [0.5:0.95] result, respectively). The reason why our model’s accuracy improvement is lower than both models is as follows. Because MS COCO images are more similar to the actual environment than PASCAL VOC (many small objects and general objects in a single image), it is difficult for the network to capture important regions. In this reason, the classification accuracy of the backbone network affects the performance improvement range. We used VGG, which achieved a lower classification accuracy than ResNet-101 and DenseNet-S-32-1, as the backbone; thus, the performance improvement is insufficient compared to other SSD-based models. Unlike the VOC dataset, RefineDet and RefineDet++ achieved high accuracies by changing the backbone from VGG to ResNet-101. However, we experimentally demonstrated that our model achieved faster detection speed as shown in Table 2.

Comparing DETR and EfficientDet with the SSD-based models including our model, the results are fairly unsatisfactory. DETR scales the input image such that the shortest side is at least 480 and at most 800 pixels, the longest side at most 1333 pixels, and EfficientDet scales to 1536×1536. Owing to the limitation of SSD’s inherent architecture, it is inferior to the state-of-the-art models. In particular, EfficientDet considerably improved the performance by utilizing an effective backbone called EfficientNet.

### 4.4. Ablation Studies

**Figure 6:**
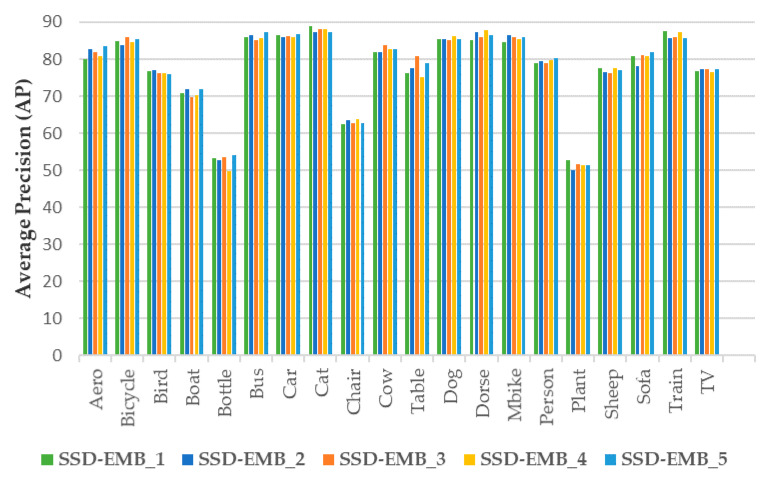
Average precision (in %) of each class for different EMBs on the PASCAL VOC 2007 test set. For SSD-EMB_1, EMB was applied to Conv4_3. For SSD-EMB_2, EMB was applied to Conv4_3 and Conv7. For SSD-EMB_3, EMB was applied to Conv7. For SSD-EMB_4, EMB was applied to Conv8_2. For SSD-EMB_5, EMB was applied to Conv4_3, Conv7, and Conv8_2.

We conducted ablation experiments to compare the effectiveness on the number of EMBs applied to the SSD and effectiveness on each stream. In the experiments, the detection accuracy of the model was compared according to the number of EMBs and the use of two streams using the PASCAL VOC 2007 test set. EMB was added after each of the Conv4_3, Conv7, and Conv8_2 layers. The experimental results are presented in Table 5 and Figure 6.

Based on Table 5 and Figure 6, the model (SSD300-EMB_5) that added three EMBs and employed both the attention and concatenation streams achieved the highest mAP. We applied EMB only on the feature maps of the shallow layers that contained low-level semantic features. In this way, the highest improvement of 1.2% was achieved. In addition, the performance achieved when the attention and feature map concatenation streams were removed one-by-one from SSD300-EMB_5 was compared. The SSD300-Attn with the use of the attention stream only achieved a mAP of 77.7%; the SSD300-Concat which applied only the concatenation stream achieved 77.9% mAP. In other words, when only one stream was used, the performance was decreased. The highest performance was achieved when the two streams were used together. Therefore, the SSD300-EMB_5 was selected as the best model based on the comparison of the detection accuracy.

In addition, we conducted an ablation experiment to compare the selection rule for the feature map split. In the feature map concatenation stream, we split the input feature map into 1/4, 2/4, 3/4, and 4/4, respectively (4/4 means that the feature map was not split), and applied three convolutions to it. The accuracy of each model was evaluated using PASCAL VOC 2007 test set. As in the above experiment, EMB was applied to Conv4_3, Conv7, and Conv8_2 layers. We presented the experimental results in Table 6.

The highest accuracy was achieved when the input feature map was split in half. Since the difference in decimal point of FPS was meaningless, we determined the selection rule that splits the input feature map in half, considering only the mAP. All EMBs applied to the model contain an attention stream and a feature map concatenation stream.

## 5. Conclusions and Future Work

The proposed EMB method used two streams, namely, attention and feature map concatenation streams. In the attention stream, we produced the 1D importance map by compressing the input feature map. In this way, the model focuses on the region wherein the small objects (or the whole objects) could exist without additional learning, and the localization accuracy was improved. In the feature map concatenation stream, one of the input feature maps (cut in half) was passed through three convolutional layers and was concatenated with the other. Based on this stream, the classification accuracy was improved. The EMB lets the high-resolution feature map of the shallow layer focus on the object regions while the accuracy of detecting small objects increases. The high accuracy of small object detection is expected to be used for satellite photo and traffic analysis. To evaluate the performance of the proposed method, the SSD-EMB was compared with the SSD [15], some SSD-based models, and state-of-the-art models. In the PASCAL VOC 2007 and 2012 benchmark datasets, the SSD-EMB enhanced the feature map of the shallow layer to improve the small object accuracy and overall accuracy. Additionally, it can be used in real-time detection. We further conducted an experiment on MS COCO. Through this experiment, we found that the performance of the model’s backbone considerably affects the overall accuracy.

In EMB, the attention stream simply focuses on the object region. In this way, localization accuracy is improved, but there is no significant effect on the classification accuracy. In this study, EMB was applied only to the SSD, but in the future work, we plan to evaluate the performance by applying the EMB to other state-of-the-art networks and will conduct research to further improve small object detection by creating a new attention mechanism and low-rank approximation that improve the classification accuracy. In our model, we utilized the fully annotated images to train the model. A fully annotated image has the class label and coordinates of the objects. However, it is a very difficult and challenging task to obtain or create these images in real life. Therefore, weakly supervised object detection, which trains model with weakly annotated images easily obtained on the web, is the focus of our future work.

## Figures and Tables

**Table 1:** Average precision (in %) for our method and others on PASCAL VOC 2007 test set. Values in bold refer to the highest average precision for each class (SSD300: single-shot multibox detector, DSSD321: deconvolutional SSD, TSEFFM: trident and squeeze and extraction feature fusion, EMB: enhanced feature map block).

Model	mAP	Aero	Bike	Bird	Boat	Bottle	Bus	Car	Cat	Chair	Cow	Table	Dog	Horse	Mbike	Person	Plant	Sheep	Sofa	Train	TV
SSD300	77.2	83.4	85.2	75.0	71.2	50.8	85.1	86.1	87.0	61.4	80.9	76.5	84.1	87.1	83.6	78.3	47.8	73.5	77.1	83.2	76.1
DSSD321	78.6	81.9	84.9	80.5	68.4	53.9	85.6	86.2	88.9	61.1	83.5	78.7	**86.7**	**88.7**	**86.7**	79.7	51.7	78.0	80.9	87.2	**79** **.4**
SSD300-TSEFFM	78.6	81.6	**94.6**	79.1	72.1	50.2	86.4	86.9	**89.1**	60.3	**85.6**	75.7	85.6	88.3	84.1	79.6	54.6	82.1	80.2	87.1	79.0
RefineDet320	**80.0**	**83.9**	85.4	**81.4**	**75.5**	**60.2**	86.4	**88.1**	**89.1**	62.7	83.9	77.0	85.4	87.1	**86.7**	**82.6**	**55.3**	**82.7**	78.5	**88.1**	**79.4**
SSD300-EMB	78.4	83.6	85.5	75.9	71.9	54.2	**87.4**	86.8	87.2	**62.8**	82.8	**78.8**	85.4	86.4	85.8	80.2	51.5	76.9	**81.9**	85.7	77.3
SSD512	79.5	84.8	85.1	81.5	73.0	57.8	87.8	88.3	87.4	63.5	85.4	73.2	86.2	86.7	83.9	82.5	55.6	81.7	79.0	86.6	80.0
DSSD513	81.5	86.6	86.2	82.6	74.9	62.5	89.0	88.7	88.8	65.2	87.0	78.7	88.2	89.0	87.5	83.7	51.1	**86.3**	81.6	85.7	83.7
SSD512-TSEFFM	80.4	84.9	86.7	80.6	76.2	59.4	87.8	88.9	89.2	61.7	86.9	**78.3**	86.2	88.8	85.6	82.7	55.4	82.7	79.4	84.7	81.3
RefineDet512	81.8	**88.7**	**87.0**	83.2	76.5	68.0	88.5	88.7	89.2	66.5	**87.9**	75.0	86.8	89.2	**87.8**	84.7	56.2	83.2	78.7	**88.1**	**82.3**
SSD512-EMB	80.4	85.9	86.2	81.3	**76.8**	59.6	87.1	88.4	88.2	67.5	85.3	76.8	87.1	**89.3**	84.9	83.0	54.2	81.6	78.7	87.8	78.3

**Table 2:** Detection results on the PASCAL visual object classes (VOC) 2007 test set (GPU: graphics processing unit, mAP: mean average precision, FPS: frames per second).

Model	Data	Backbone Network	Input Size	GPU	Framework	#Parameters	mAP	FPS
SSD300*	07 + 12	VGG	300×300	2080Ti	PyTorch	26.3 M	77.2	30
SSD300* ^1^	07 + 12	VGG	300×300	Titan X	Caffe	26.3 M	77.2	46
DSSD321	07 + 12	ResNet-101	321×321	Titan X	Caffe	- ^2^	78.6	9.5
SSD300-TSEFFM	07 + 12	VGG	300×300	2080Ti	PyTorch	-	78.6	7
DF-SSD300	07 + 12	DenseNet-S-32-1	300×300	Titan X	Caffe	15.2 M	78.9	11.6
RefineDet320	07 + 12	VGG	320×320	Titan X	Caffe	-	80.0	40.3
RefineDet320++	07 + 12	VGG	320×320	Titan X	PyTorch	-	81.1	27.8
SSD300-EMB	07 + 12	VGG	300×300	2080Ti	PyTorch	30.6 M	78.4	30

^1^ SSD300 was tested with the PyTorch deep learning framework and RTX 2080Ti GPU, while SSD300* was tested with the Caffe deep learning framework and Titan X GPU. ^2^ All data not mentioned in their papers are marked with ‘-’.

**Table 3:** Average precision (in %) for our method and others on the PASCAL VOC 2012 test set. Bold indicates the highest average precision for each class.

Model	mAP	Aero	Bike	Bird	Boat	Bottle	Bus	Car	Cat	Chair	Cow	Table	Dog	Horse	Mbike	Person	Plant	Sheep	Sofa	Train	TV
SSD300	75.8	88.1	82.9	74.4	61.9	47.6	82.7	78.8	91.5	58.1	80.0	64.1	89.4	85.7	85.5	82.6	50.2	79.8	73.6	86.6	72.1
DSSD321	76.3	87.3	83.3	75.4	64.6	46.8	82.7	76.5	92.9	59.5	78.3	64.3	**91.5**	86.6	86.6	82.1	**53.3**	79.6	**75.7**	85.2	**73.9**
DF-SSD300	76.5	89.5	85.6	72.6	65.8	51.3	82.9	79.9	92.2	**62.4**	77.5	64.5	89.5	85.4	86.4	**85.7**	51.9	77.8	72.6	85.1	71.6
SSD300-TSEFFM	77.1	88.6	**85.9**	76.0	65.4	46.2	**84.0**	79.9	**92.7**	58.6	81.9	65.3	**91.5**	**87.8**	**88.8**	82.9	52.6	79.1	75.4	87.1	73.8
RefineDet320	78.1	**90.4**	84.1	**79.8**	**66.8**	**56.1**	83.1	**82.7**	90.7	61.7	**82.4**	63.8	89.4	86.9	85.9	**85.7**	**53.3**	**84.3**	73.1	87.4	**73.9**
SSD300-EMB	77.0	88.8	85.4	75.4	63.6	50.3	83.5	79.4	92.1	59.5	81.4	**66.2**	88.9	86.6	86.3	83.3	51.5	80.5	75.6	**88.1**	73.3
SSD512	78.5	90.0	85.3	77.7	64.3	58.5	85.1	84.3	92.6	61.3	83.4	65.1	89.9	88.5	88.2	85.5	54.4	82.4	70.7	87.1	75.6
DSSD513	80.0	**92.1**	86.6	80.3	68.7	58.2	84.3	85.0	**94.6**	**63.3**	85.9	65.6	**93.0**	88.5	87.8	86.4	57.4	85.2	**73.4**	87.8	**76.8**
SSD512-TSEFFM	80.2	90.1	88.2	81.5	68.4	59.1	85.6	**85.5**	93.7	63.0	**86.1**	64.0	90.9	88.6	89.1	86.4	59.2	85.9	73.3	87.8	75.9
RefineDet512	80.1	90.2	86.8	**81.8**	68.0	**65.6**	84.9	85.0	92.2	62.0	84.4	64.9	90.6	88.3	87.2	**87.8**	58.0	**86.3**	72.5	**88.7**	76.6
SSD512-EMB	79.9	90.2	**88.5**	78.4	67.7	59.5	**86.2**	84.7	93.1	62.7	84.5	**67.1**	91.3	88.9	**89.5**	86.1	58.1	84.3	73.0	87.8	76.3

**Table 4:** Average precision (in %) for our method and others on MS COCO test-dev 2015. Bold indicates the highest value for each column. Avg. Precision, IoU corresponds to the average APs for IoU (0.5~0.95, 0.5, 0.75). AP@[0.5:0.95] is performed with a step size of 0.05. Avg. Precision, Area means the average APs for the object area size (S, M, L). Avg. Recall, #Dets means the average ARs for the number of detections (1, 10, 100). Avg. Recall, Area indicates the average ARs for the object area size (S, M, L).

Model	Data	Network	Avg. Precision, IoU:			Avg. Precision, Area:			Avg. Recall, #Dets:			Avg. Recall, Area:		
			0.5:0.95	0.5	0.75	S	M	L	1	10	100	S	M	L
SSD300	trainval35k	VGG	25.1	43.1	25.8	6.6	25.9	41.4	23.7	35.1	37.2	11.2	40.4	58.4
DSSD321	trainval35k	ResNet-101	28.0	46.1	29.2	7.4	28.1	47.6	25.5	37.1	39.4	12.7	42.0	62.6
DF-SSD300	trainval	DenseNet-S-32-1	29.5	50.7	31.3	9.8	31.1	46.5	27.1	41.5	42.7	17.3	46.8	64.4
RefineDet320	trainval35k	ResNet-101	32.0	51.4	34.2	10.5	34.7	50.4	28.0	44.0	47.6	20.2	53.0	69.8
RefineDet320++	trainval35k	ResNet-101	33.2	53.4	35.1	13.1	35.5	51.0	28.3	44.5	**47.8**	**20.9**	**53.1**	**70.1**
DETR	trainval	ResNet-101	44.9	64.7	47.7	**23.7**	**49.5**	**62.3**	- ^1^	-	-	-	-	-
EfficientDet	train	EfficientNet	**55.1**	**74.3**	**59.9**	-	-	-	-	-	-	-	-	-
SSD300-EMB	train	VGG	26.6	45.2	27.8	7.3	29.3	43.5	25.4	36.4	38.6	12.0	41.7	60.3

^1^ All data not mentioned in their papers are marked with ‘- ’.

**Table 5:** Influences of EMB when applied on the PASCAL VOC 2007 test set. Bold indicates our model adopted by the ablation study.

Model	Conv4_3	Conv7	Conv8_2	Attention stream	Concatenation stream	mAP
SSD300						77.2
SSD300-EMB_1	✔			✔	✔	77.8
SSD300-EMB_2	✔	✔		✔	✔	77.9
SSD300-EMB_3		✔		✔	✔	78.1
SSD300-EMB_4			✔	✔	✔	77.8
SSD300-Attn	✔	✔	✔	✔		77.7
SSD300-Concat	✔	✔	✔		✔	77.9
SSD300-EMB_5	✔	✔	✔	✔	✔	**78.4**

**Table 6:** Influences of feature map split on the PASCAL VOC 2007 test set. Bold indicates our model adopted by the ablation study.

Model	1/4	2/4	3/4	4/4	FPS	mAP
SSD300-EMB_1/4	✔				30.7	77.9
SSD300-EMB_2/4		✔			**30.4**	**78.4**
SSD300-EMB_3/4			✔		30.2	78.1
SSD300-EMB_4/4				✔	30.0	78.3

## Data Availability

Publicly available datasets were analyzed in this study. This data can be found here: https://github.com/HTCho1/SSD-EMB.Pytorch/ (accessed on 14 April 2021).
